# Towards traceable size determination of extracellular vesicles

**DOI:** 10.3402/jev.v3.23298

**Published:** 2014-02-04

**Authors:** Zoltán Varga, Yuana Yuana, Anita E. Grootemaat, Edwin van der Pol, Christian Gollwitzer, Michael Krumrey, Rienk Nieuwland

**Affiliations:** 1 Department of Biological Nanochemistry, Institute of Molecular Pharmacology, Research Centre for Natural Sciences, Hungarian Academy of Sciences, Budapest, Hungary; 2 Department of Clinical Chemistry, Academic Medical Centre of the University of Amsterdam, Amsterdam, The Netherlands; 3 Department of Biomedical Engineering and Physics, Academic Medical Centre of the University of Amsterdam, Amsterdam, The Netherlands; 4 Physikalisch-Technische Bundesanstalt (PTB), Berlin, Germany

**Keywords:** exosome, microvesicle, erythrocyte, extracellular vesicles, freeze-fracture transmission electron microscopy, nanoparticle tracking analysis, resistive pulse sensing, small-angle X-ray scattering, dynamic light scattering, size exclusion chromatography

## Abstract

**Background:**

Extracellular vesicles (EVs) have clinical importance due to their roles in a wide range of biological processes. The detection and characterization of EVs are challenging because of their small size, low refractive index, and heterogeneity.

**Methods:**

In this manuscript, the size distribution of an erythrocyte-derived EV sample is determined using state-of-the-art techniques such as nanoparticle tracking analysis, resistive pulse sensing, and electron microscopy, and novel techniques in the field, such as small-angle X-ray scattering (SAXS) and size exclusion chromatography coupled with dynamic light scattering detection.

**Results:**

The mode values of the size distributions of the studied erythrocyte EVs reported by the different methods show only small deviations around 130 nm, but there are differences in the widths of the size distributions.

**Conclusion:**

SAXS is a promising technique with respect to traceability, as this technique was already applied for traceable size determination of solid nanoparticles in suspension. To reach the traceable measurement of EVs, monodisperse and highly concentrated samples are required.

Exosomes, microparticles and other extracellular vesicles (EVs) have gained particular attention due to their role in biological processes ranging from intercellular communication and angiogenesis to cell survival (1–7). Throughout this manuscript, we will use the term “EVs” to refer to all classes of cell-derived extracellular vesicles. Since EVs are associated to many pathological conditions such as thrombosis, haemostasis, inflammation, sickle cell disease and malaria (8–11), they may serve as biomarkers of disease and therapeutic targets (12). However, due to the difficulties of isolation and detection of vesicles (13–16), standardization is not yet achieved (17).

Isolating EVs from body fluids is affected by collection, handling and the presence of similar-sized contaminants, such as protein complexes/aggregates and lipoproteins (13). Although differential centrifugation protocols are frequently used for the isolation of EVs, it involves some practical problems. For example, due to the high centrifugal forces, cells may fragment or become activated leading to artefactual release of vesicles, and proteins and EVs also may aggregate (14). Moreover, EV samples isolated by differential centrifugation may still contain lipoproteins and protein complexes (14, 15), thereby affecting size determination of EVs and also characterization of EVs using techniques such as western blot or proteomics (3, 11, 18–21).

The determination of the size distribution of EVs is important from the point of vesicle classification and especially needed for choosing the best method to accurately measure EVs. Size characterization of EVs is challenging due to their small size and heterogeneity (16, 22). The diameter of vesicles typically ranges from 1 to 30 nm, which is below the detection range of common methods such as conventional flow cytometer (16). In addition, many new techniques have not been validated yet. Consequently, there is a need for traceable size determination of EVs. “Traceable size determination” means that the measurement result can be related to the SI unit “metre” through an unbroken chain of comparisons with known uncertainties (23, 24). Traceable measurements of EVs can be used to develop reference materials with similar properties to EVs and to calibrate other detection methods, thereby facilitating standardization.

In this study, 5 different methods were applied to characterize erythrocyte-derived EVs. Two methods, namely small-angle X-ray scattering (SAXS) and size exclusion chromatography coupled with on-line dynamic light scattering detection (SEC-DLS), were used for the first time to detect EVs. In addition, we studied the purity of EVs using these 2 novel techniques. For comparison, the same population of EVs was characterized by freeze-fracture transmission electron microscopy (FF-TEM), nanoparticle tracking analysis (NTA) and resistive pulse sensing (RPS). NTA and RPS are relatively new techniques used to determine concentration and size distribution of EVs (25–27), whereas FF-TEM is not described in the current literature but has been used for characterization of vesicles in the 1970s (28). Brief descriptions and specifications on the used techniques are given in the next section. Advantages and drawbacks of each method are also discussed, which may be used in reaching traceable characterization of EVs.

## Materials and methods

### Preparation of erythrocyte vesicles

Erythrocyte vesicles were isolated from out-dated erythrocyte concentrates, so-called packed cells (Sanquin, Amsterdam, The Netherlands). Packed cells were diluted 2-fold with phosphate buffered saline (PBS; AMC, Department of Pharmacy, Amsterdam, The Netherlands, pH 7.45). Erythrocytes were removed by 2 centrifugation steps at 1,550×g for 20 minutes at 20°C. Vesicles in the erythrocyte-free supernatant were concentrated by centrifugation at 18,890×g for 0.5 hour to obtain approximately 960-fold concentrated erythrocyte EV sample. These samples were snap-frozen in liquid nitrogen and stored at −80°C until use.

### Freeze-fracture transmission electron microscopy

In case of FF-TEM, the sample is frozen within a few milliseconds, which inhibits the crystal formation in the aqueous samples to preserve the original morphology. FF-TEM does not require fixation and negative staining, which are procedures that most likely affect the sample. Therefore, FF-TEM provides a more realistic picture about the structure of EVs in suspension than TEM (29–31). For FF-TEM, the vesicle sample was mixed with glycerol (Sigma-Aldrich, Budapest, Hungary) at 3:1 sample: glycerol volume ratio. The use of glycerol as cryoprotectant is needed to obtain homogeneously distributed vesicles. The vesicle sample (1–2 µl) was pipetted onto a gold sample holder and frozen by plunging it immediately into partially solidified Freon for 20 seconds and stored in liquid nitrogen. Fracturing was performed at −100°C in a Balzers freeze-fracture device (Balzers BAF 400D, Balzers AG, Vaduz, Liechtenstein). The replicas of the fractured faces etched at −100°C were made by platinum–carbon shadowing and then cleaned with a water solution of surfactant and washed with distilled water. The replicas were placed on 200 mesh copper grids and examined in a MORGAGNI 268D (FEI, The Netherlands) transmission electron microscope.

### Nanoparticle tracking analysis

NTA is widely used in vesicle research for the characterization of the size and concentration of EVs (27). With NTA, the Brownian motion of individual particles in solution is tracked based on their light scattering. Variables include the camera level, detection threshold, viscosity, temperature, and the dilution of the sample. There are some attempts to standardize this method with regard to characterization of EVs (27, 32). An NS500 (NanoSight Limited, London, UK) equipped with an electron multiplying charge coupled device camera (Andor Technology, Tokyo, Japan) and a 405 nm laser was used. During measurements, temperature was kept at 22°C. The viscosity of water at 22°C (0.95 mPa s) was used, as samples were diluted several fold in PBS buffer. Silica beads with a diameter of 100 nm and known concentration were used to adjust the focus height of the objective and to calibrate the concentration (27). NTA v2.3.0.17 software (NanoSight Limited) was used for data analysis. Before the measurement, samples were 500,000-fold diluted with PBS buffer. Ten videos of 30 seconds were captured per measurement at camera level 15. The detection threshold was set at pixel value 16.

### Resistive pulse sensing

RPS is capable of determining the size and concentration of EVs based on the Coulter principle (22, 25, 33). An RPS instrument (qNano, Izon Science Ltd., Christchurch, New Zealand) equipped with an NP100A type membrane (particle detection range: 70–200 nm) was used to measure the size distribution and concentration of EVs in suspension. RPS was operated at a voltage of 0.50 V and a pressure of 12 cm H_2_O. The membrane was stretched at 47.0 mm. Polystyrene beads with a concentration of 1.0×10^10^ beads/mL (115 nm; Izon Science) were used to calibrate the size and concentration following manufacturer's instructions. Samples were diluted 10,000-fold with PBS buffer and measured for 10 minutes.

### SEC combined DLS

With size exclusion chromatography (SEC) combined with DLS, particles and macromolecules are separated by their size and subsequently measured by DLS. A SEC-based isolation protocol has been already used to purify EVs (34, 35), but so far it was not used for the detection of EVs. On the other hand, SEC is widely used for the characterization of liposomes, which have the same size and morphology as EVs (36, 37).

A high-precision liquid chromatography (HPLC) column filled with a macroporous hydroxylated methacrylate gel (TSK G6000PW column, Tosoh Corp., Tokyo, Japan; mean particle size 17 µm, mean pore size >100 nm) was used, which can separate phospholipid liposomes in the size range between 30 and 200 nm and macromolecules in the molecular weight range of 40–8,000 kDa (36). For the SEC measurements, the sample was centrifuged at 5,000×g for 10 minutes to remove possible aggregates that may have formed during thawing and may clog the column. The supernatant was analysed with a Jasco HPLC system (Jasco, Tokyo, Japan) consisting of a PU-2089 pump with UV-2075 UV/Vis detector and Rheodyne 7725i injector controlled by the Chromnav software v. 1.17.02. On-line DLS detection was performed by a W130i DLS instrument (Avid Nano Ltd., High Wycombe, UK) directly coupled to the HPLC system using a flow-through cuvette (Type No. 176.753-QS, Hellma Analytics, Müllheim, Germany). PBS was used as eluent at 1 mL/min flow rate, and ultraviolet (UV) detection was performed at 210 nm wavelength. The scattering intensity at 90° and the autocorrelation function accumulated for 3 seconds was measured with the DLS setup equipped with a 660 nm laser. The autocorrelation function is the cross-correlation of the scattered intensity signal with itself, and carries information about the size of the particles because of the Brownian motion. If the sample is monodisperse, the autocorrelation function is a simple exponential decay, with an exponent proportional to the hydrodynamic diameter. In case of a polydisperse sample, the autocorrelation function becomes the sum of exponential decays each corresponding to a subspecies of the distribution. In the latter case, different mathematical algorithms are used to obtain the size distribution of the sample. The on-line DLS data were processed with the iSize 2.0 software (Avid Nano Ltd., High Wycombe, UK). To demonstrate the effect of SEC on the DLS results, measurements were performed off-line using a low-volume disposable cuvette (UVette, Eppendorf Austria GmbH). Size distributions from the DLS data were calculated using the SEDFIT software (Peter Schuck, National Institutes of Health, Bethesda, MD, USA http://www.analyticalultracentrifugation.com), utilizing the CONTIN algorithm (38).

### Small-angle X-ray scattering

SAXS can provide structural information on nanomaterials in the 1–200 nm size range. SAXS is based on the elastic scattering of X-ray photons on the electrons of the sample at low angles. The scattering intensity is measured as a function of the momentum transfer *q*, which is related to half of the scattering angle θ between the direction of the incident beam and the scattered light according to *q*=4π/λ sin(θ), where *λ* denotes the wavelength of the incident X-ray beam. The structural features of the sample with a size of *d* are indirectly represented in the scattering curve by the intensity at *q*=2π/*d*, and can be extracted by fitting theoretical models to the measured data. SAXS was already applied to describe different vesicle systems from synthetic (39–41) and natural origin (42), but its use in characterization of EVs is unprecedented.

SAXS measurements were performed at the four-crystal monochromator beamline (43) of Physikalisch-Technische Bundesanstalt (PTB) supplemented by the SAXS setup of Helmholtz-Zentrum Berlin (44) at the synchrotron radiation facility BESSY II (Helmholtz-Zentrum Berlin, Germany). The measurements were carried out using 2 different experimental set-ups to cover the size range from about 200 to 2 nm. This requires covering the range of momentum transfer *q* from 0.015 to 2.5 nm^−1^.

In the first setup, used to access the *q*-range describing the overall size of the vesicles, the sample-to-detector distance was 4.4 m and the energy of the incoming X-ray beam was set to 4 keV (wavelength 0.31 nm). Due to the short penetration length of X-rays at this energy, a custom-made sample holder was used utilizing silicon-nitride windows (NX7150E, Norcada Inc., Edmonton, Canada). In the second setup, used to characterize objects down to 2 nm, the sample-to-detector distance was 2.2 m, the X-ray energy was set to 10 keV (wavelength 0.124 nm) and the sample was filled into borosilicate glass capillaries with 1.0 mm nominal diameter (Müller & Müller OHG, Berlin, Germany).

For both setups, 2D scattering patterns were collected with a vacuum-compatible large-area pixel detector Pilatus 1M (Dectris Ltd., Baden, Switzerland) (45). All measurements were performed at room temperature. The scattering curves were obtained by radial averaging of the 2D patterns. Non-linear least squares fitting of an empirical model function to the scattering curves was performed using the SASfit program v. 0.93.3 (Joachim Kohlbrecher and Ingo Bressler, Paul Scherrer Institute, Villigen, Switzerland). The applied model function can be found in the Supplementary file.

## Results

### Size distribution by established methods

**Fig. 1:**
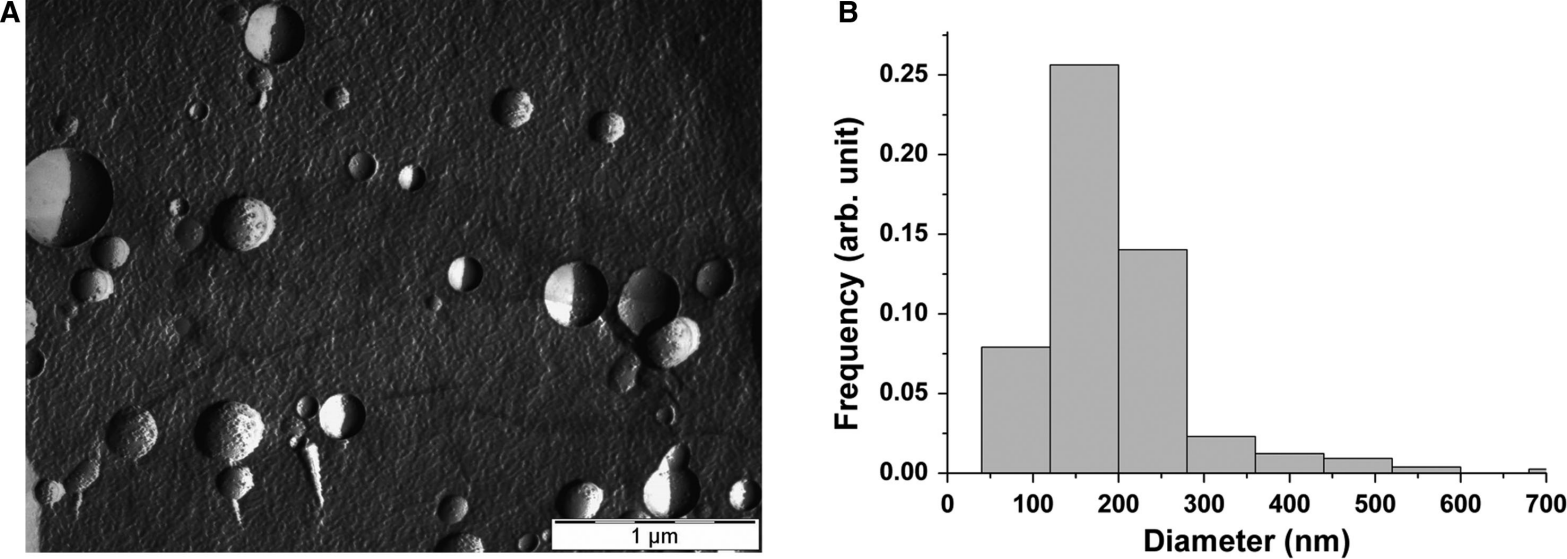
(A) Typical FF-TEM picture of the erythrocyte EVs. (B) The number weighted size distribution based on the FF-TEM images of erythrocyte EVs.

Figure 1A shows a typical FF-TEM picture of the platinum–carbon freeze-fracture replicas of the studied erythrocyte EVs. Spherical vesicles in the size range of 50–300 nm can be clearly observed on the FF-TEM picture (Fig. 1A). The apparent diameter from the pictures results in the so-called “Freeze-fracture” weighted size distribution, as the fracturing of a vesicle takes place at a diameter less than or equal to the maximum diameter. By using a transformation based on geometrical considerations, the number weighted distribution can be obtained (46). This is shown in Fig 1B. For the construction of the distribution, at least 300 individual vesicles were counted. The distribution obtained by FF-TEM has a maximum corresponding to the 120–200 nm size class, while vesicles with diameters up to 500 nm are also present in the size distribution.

**Fig. 2:**
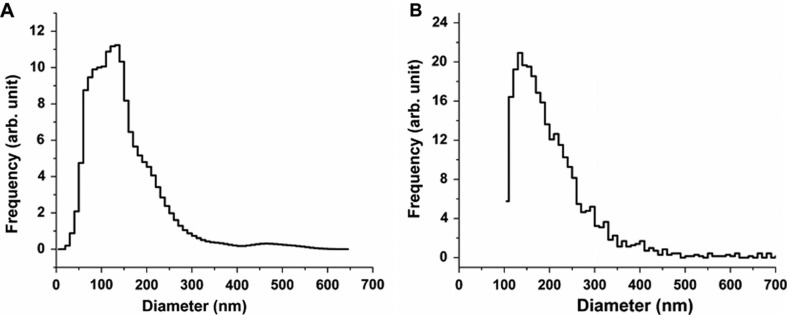
Size distributions of the erythrocyte EVs obtained by NTA (A) and RPS (B).

Figure 2A shows the size distribution of the studied erythrocyte EVs obtained by NTA. The features of the size distribution are similar to those from FF-TEM, though NTA detects a large number of vesicles in the sub-100 nm size range. The mode diameter and the full width at half maximum (FWHM) of the distribution obtained by NTA is 135 and 114 nm, respectively.

Figure 2B shows the distribution of the studied erythrocyte EVs obtained by RPS. The size distribution measured with the NP100A membrane shows a lower cut-off at 100 nm. The mode diameter of the distribution obtained by RPS is 135 nm, which corresponds well with that obtained by NTA. Because of the truncation of the distribution at 100 nm, determination of the FWHM is ambiguous.

Both NTA and RPS are single particle counting methods, and are capable of determining the particle concentration. We obtained 1.6×10^13^ and 2.6×10^13^ particles/mL by NTA and RPS, respectively, but we observed large deviations by using different settings in both cases, so the distributions shown here are displayed in arbitrary units. The characterization of the concentration of EVs is out of the scope of this manuscript.

### Size distribution and purity by SEC-DLS and SAXS

The previously discussed techniques provide no information on the purity of EVs concerning protein contamination. Protein contamination is relevant with regard to subsequent analysis, such as proteomics (3, 18, 19, 21). To obtain reliable information about the amount of contaminating plasma proteins in the vesicle preparations, SEC investigations were carried out. Supplemented with on-line DLS detection, this approach was also used for sizing EVs.

**Fig. 3:**
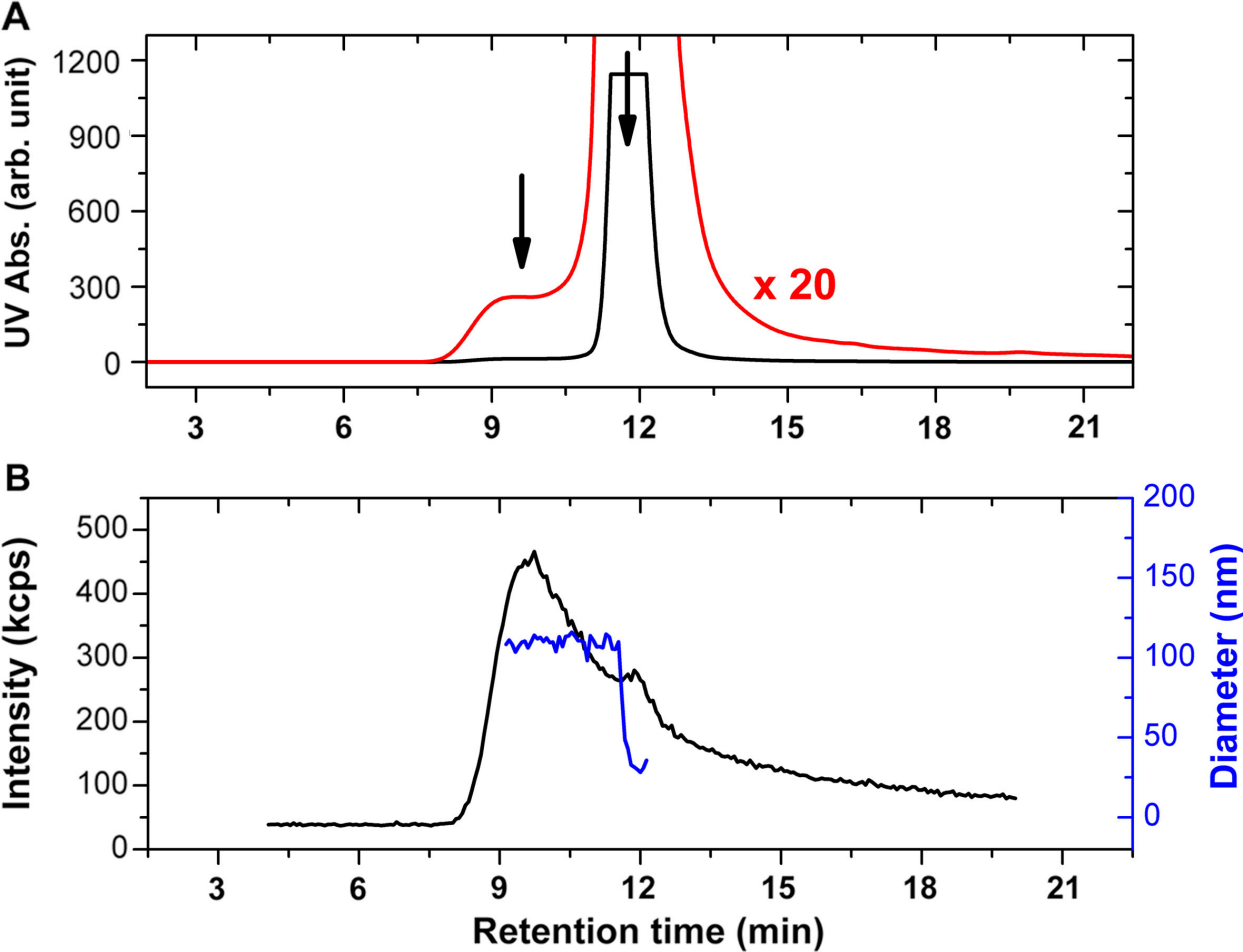
HPLC-SEC-DLS results of the erythrocyte EVs. The UV absorption signal at 210 nm for the studied sample is shown in A (black line), together with the magnification of the signal by a factor of 20 (red line). The scattered intensity at 90° of the DLS detector is shown in (B) (black line) together with the mean diameter obtained from autocorrelation data (blue line). The fractions related to the vesicles and the plasma proteins can be clearly identified at 9.6 and 11.9 minutes elution times, respectively (indicated by arrows). The signal of the plasma proteins in case of the UV detection almost completely overwhelms that of the vesicles, but the situation is reversed in case of the DLS detection, where the light scattering from the vesicles is dominating, and the plasma proteins are represented only by a small-side peak.

Figure 3A shows the UV absorption signal versus retention time for the EV sample. The chromatogram is dominated by the contribution from the free proteins at 11.9 minutes elution time close to the total volume of the used column. A closer magnification on the UV signal shows a broad peak centred at 9.6 minutes which can be attributed to the vesicle fractions based on the retention time of liposomes with size similar to the investigated EV sample (36). Figure 3B shows the scattered intensity at 90**°** of the DLS detector. According to the large difference between the scattering intensity of vesicles and plasma proteins, the light scattering detection clearly identifies the vesicle fraction at 9.6 minutes elution time and the plasma protein fraction as a side peak with relatively low intensity. The results of the cumulant analysis of the individual correlation functions are also shown in Fig. 3B, which shows a nearly constant average size for the elution peak of the vesicles.

**Fig. 4:**
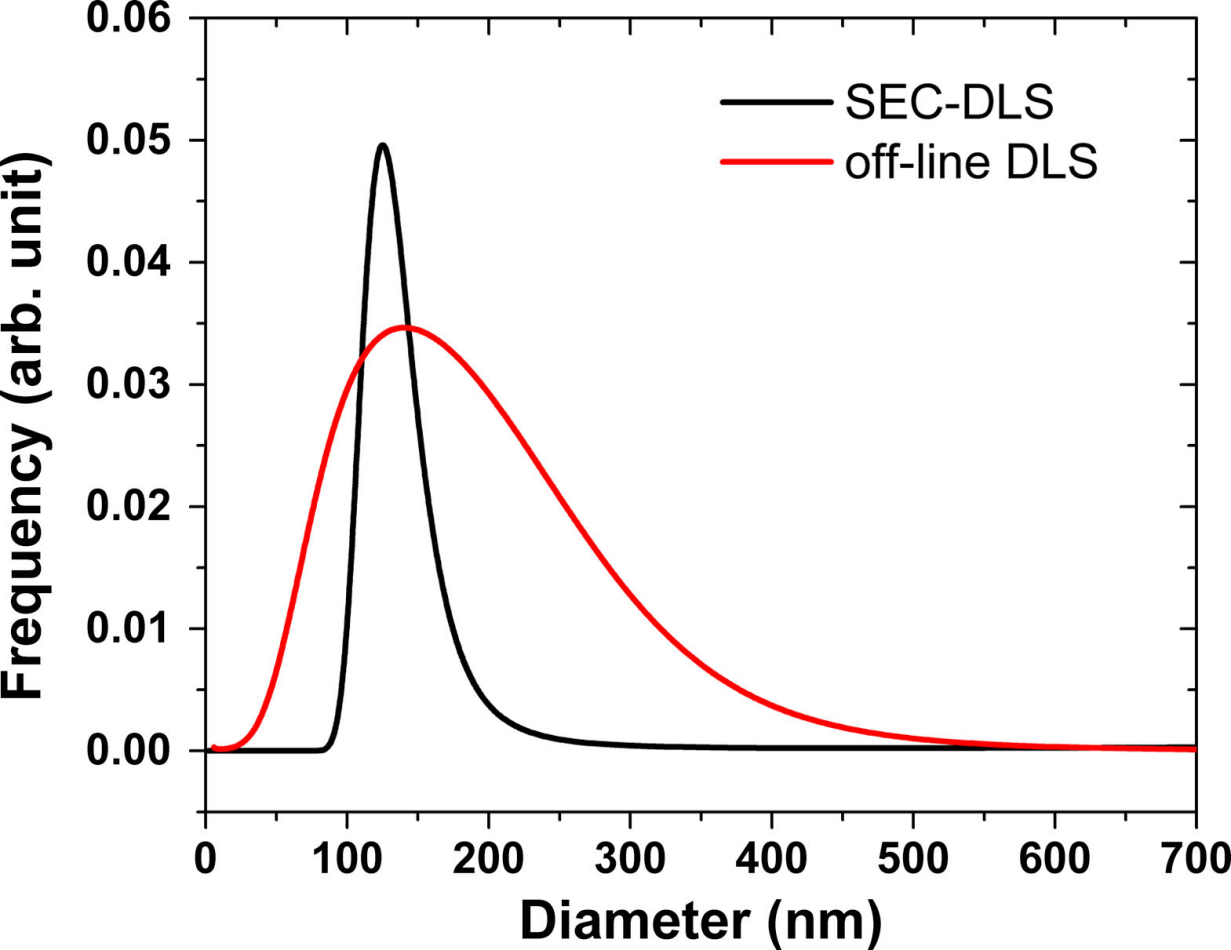
DLS results of the erythrocyte EVs. Intensity weighted size distributions obtained by the CONTIN algorithm are shown for the samples measured on-line during SEC (black line) and off-line (red line). In case of the on-line data, the autocorrelation functions measured for 3 seconds were summed up for the elution peak corresponding to the EV fractions.

Size distributions from the on-line DLS measurements were calculated using the CONTIN algorithm. For this purpose, the autocorrelation data from the elution peak corresponding to the vesicle fraction was summed up. For comparison, the sample was also measured with off-line DLS in a low-volume cuvette. Figure 4 shows the obtained size distributions. The on-line DLS measurement indicates a mode diameter of 123 nm for the studied EV sample, while the off-line measurement in a cuvette results in 141 nm. The FWHMs of the 2 distributions are 46 and 196 nm for the on-line and the off-line measurements, respectively. On-line DLS results in a narrower size distribution and smaller mode diameter compared to off-line DLS. During SEC, the main vesicle fraction is separated from the plasma proteins as well as from the large vesicles (>500 nm) that might be present in the preparation in a sub-percent amount but can affect the DLS data evaluation significantly. This separation makes the evaluation of the autocorrelation data more reliable in case of the on-line DLS measurement, which might be the explanation for the differences between the size distributions from the on-line and off-line DLS data.

**Fig. 5:**
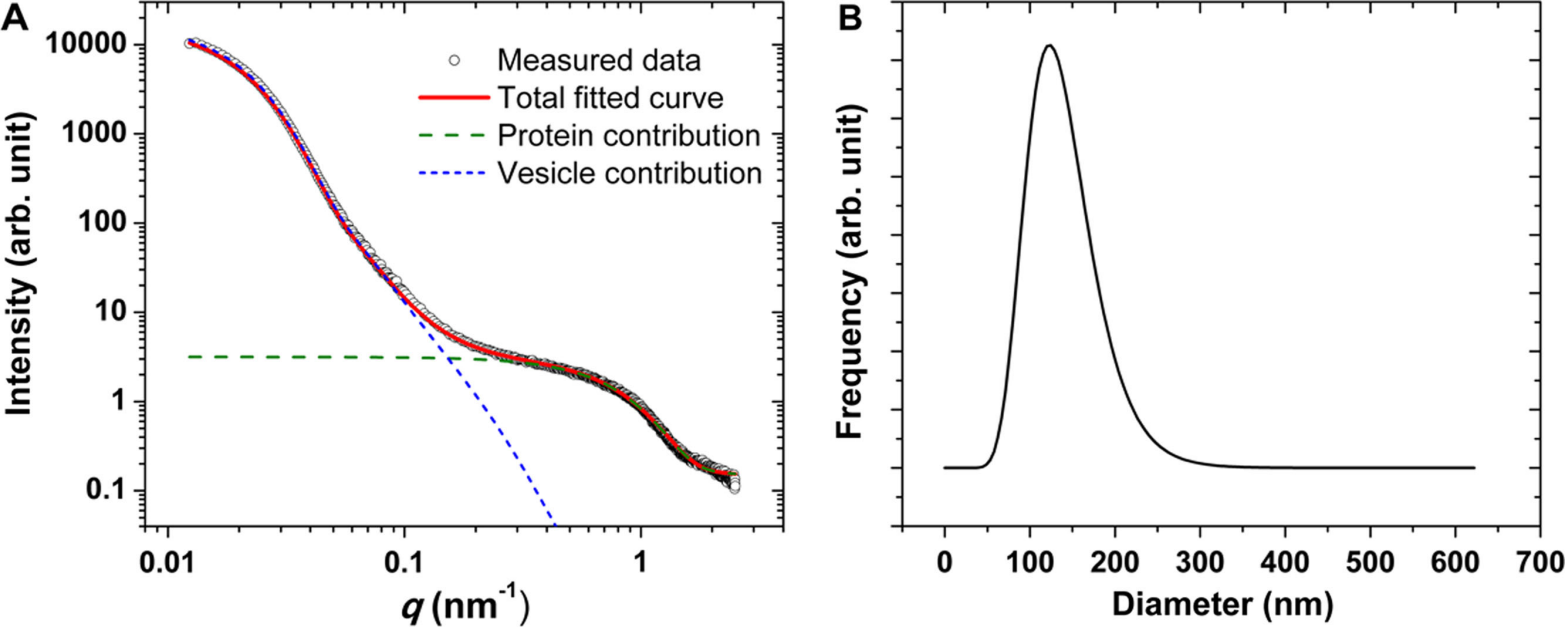
(A) The measured SAXS curve of the erythrocyte EVs (circles), together with the best fitting model function (red line). The contribution from the vesicles and the plasma proteins based on the fitted function is also highlighted (blue and green lines, respectively). (B) Number distribution of the vesicle fraction corresponding to the best fitting model function.

Figure 5 shows the results of the SAXS characterization of the studied EV sample. As discussed in the introduction, SAXS is well suited for the characterization of nanoparticles, but a traceable size determination has up to now only been demonstrated for spherical and sufficiently monodisperse nanoparticles (24, 44). Figure 5A shows the scattered intensity as the function of momentum transfer *q* for the erythrocyte EV sample. For the interpretation of the data, an analytical intensity expression was fitted, which takes into account the vesicles as core-shell particles (with a shell representing the phospholipid bilayer of the vesicles, and a core representing the aqueous inner volume containing proteins and nucleic acids), and plasma proteins as spherical particles with constant electron density. The model function fitting best to the experimental data together with the intensity contributions from the vesicle and the plasma protein fractions is also shown in Fig. 5A. The fitted model function fits well to the experimental data, which supports our assumptions. There are 2 main features in this curve: (a) The scattering from the vesicles appears at low values of *q*, that is, at very small-angles, as their size falls in the 100 nm size range; (b) The presence of free proteins can be clearly identified by the X-ray scattering in the 0.5–1.5 nm^−1^ *q*-range.

The size distribution calculated from the parameters of the model used for the vesicles is shown in Fig. 5B. This distribution has a mode value at 125 nm diameter and a FWHM of about 90 nm.

## Discussion

**Fig. 6:**
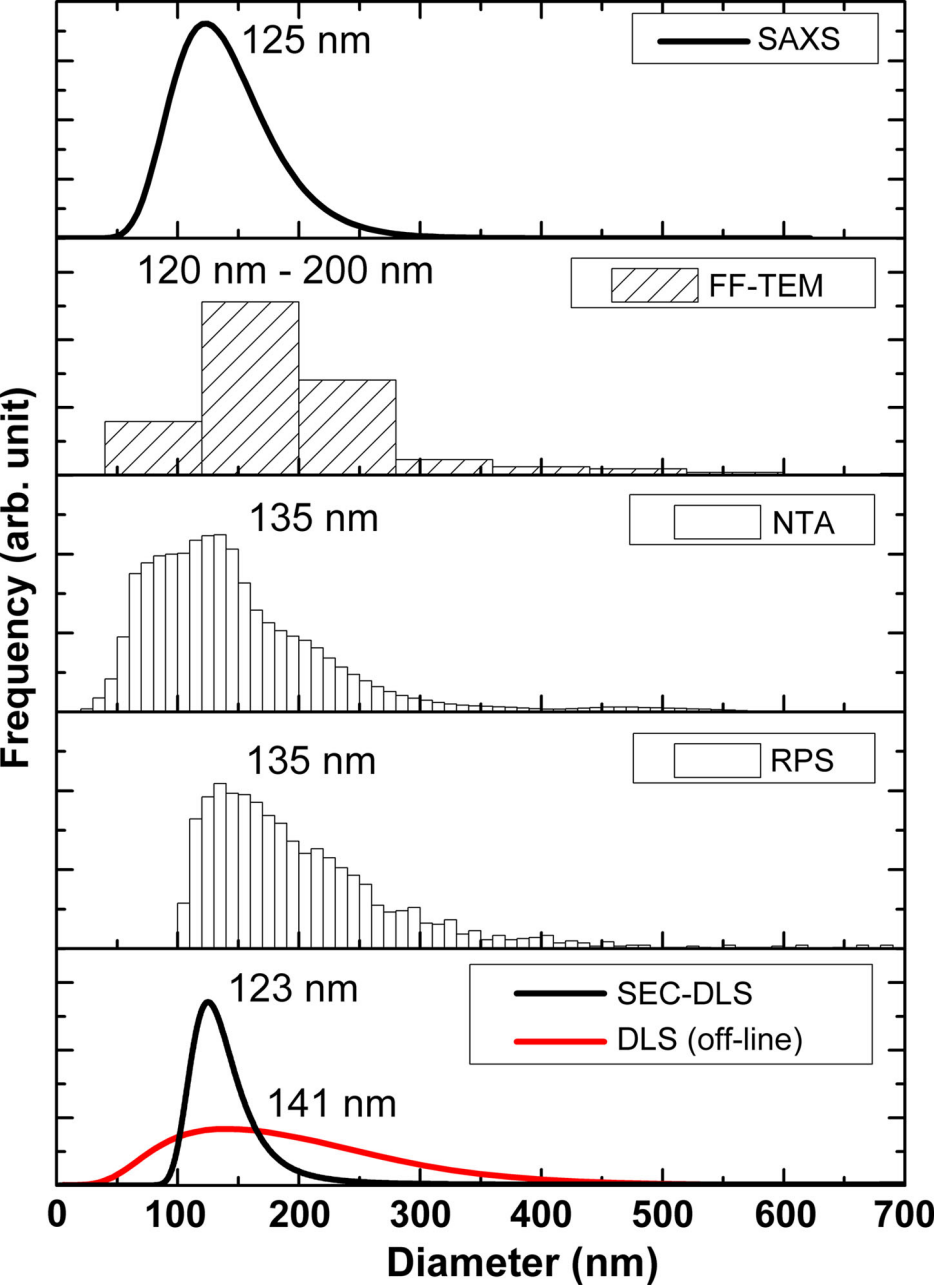
Size distributions of the erythrocyte EV sample by the applied techniques. The numbers in each subfigure indicate the mode diameter of the distribution, except for FF-TEM, where the size range of the bin with the most frequent counts is displayed.

We have characterized the size of erythrocyte-derived EVs using FF-TEM, RPS, NTA, SEC-DLS and SAXS, as shown in Fig. 6. The first noteworthy observation is that most methods also detect vesicles with a diameter below 100 nm, despite the fact that the applied g-force is believed to sediment only vesicles larger than 100 nm(e.g. microvesicles/microparticles/ectosomes) (17). RPS is an exception, because the pore size of the used membrane is insufficient for sizing vesicles with a diameter less than 100 nm. All methods, except FF-TEM, result in a distribution having a mode value between approximately 120 and 140 nm, but there are pronounced variations in the width of the distributions among the different techniques. With FF-TEM, most of the vesicles belong to the 120–200 nm size class.

For a traceable measurement of the size distribution, the length scale of the method has to be traceable to the SI unit “metre” and the sample has to fulfil certain requirements. In the following section, we discuss the techniques used in this study for the size determination of EVs with respect to their traceability.

### FF-TEM

Size determination by electron microscopy techniques can be made traceable (47) similarly to atomic force microscopy (48), but special attention needs to be made to the image processing techniques and to the statistical significance of the observed objects. The FF-TEM investigations enabled to identify the presence of EVs without fixation and dehydration, and their high concentration was sufficient to obtain the size distribution. Two important notes should be made here: (a) the concentration of the studied samples were at least 2 or 3 orders of magnitude higher than in usual EV preparations, (b) the number of vesicles counted on the pictures is still low for a reliable determination of the size distribution. The main question about the traceable size determination of EVs by electron microscopic techniques, such as FF-TEM and cryo-TEM, is whether the freezing process modifies the structure of EVs in suspension. In case of FF-TEM, the uncertainty introduced by making a replica of the fractured surface, that is, the complex process in which the non-wetting platinum covers a frozen aqueous surface, also hampers the traceability.

### NTA

The results obtained by NTA coincide well with the observations from FF-TEM. Since the number of tracked vesicles is 5 times larger than the counted vesicles on the FF-TEM pictures, the distribution from NTA can be considered to be characteristic for the ensemble. Determining the uncertainty in NTA measurements is particularly difficult because of the high number of parameters used in the data evaluation, and the lack of a suitable transfer standard for EVs. Moreover, the relevant quantity obtained by NTA is the hydrodynamic diameter through the determination of the diffusion coefficients, but the relationship between the hydrodynamic and geometrical diameter is usually unknown, and highly depends on the surface properties of the particles and the solvent.

### RPS

The mode diameter reported by RPS is also in line with the values reported by the other techniques, though the shape of the distribution by RPS clearly indicates that this technique was not able to detect a vesicle fraction below 100 nm. RPS is based on the Coulter principle, and it is independent of any optical properties as well as the viscosity of the solvent. RPS requires the particles to be non-conductive relative to the medium, a requirement that is most likely valid for vesicles. However, a traceable uncertainty analysis in case of RPS is difficult and not reached yet, due to the complex relationship between the measured current signal and volume of the particle analysed. The surface properties of the particles, the imperfect shape of the nanopore and the possible non-specific adhesion of the vesicles to the membrane contribute to the result of the measurement with unknown uncertainties.

### SEC-DLS

Analytical SEC with on-line DLS detection was also used in our study for the identification and size determination of EVs. The mode diameter of EVs obtained from the SEC-DLS measurements corresponds well with the values reported by the other techniques, which indicate that using SEC some limitations of the sizing by DLS can be overcome. The standard detection during the SEC analysis utilizing UV absorption measurement clearly identified the fractions related to vesicles and to plasma proteins, which might have further application in quality control of vesicle preparations. The principle of SEC is based only on the size of the particles, but in practice, electrostatic and steric interactions between the particles and the stationary phase always contribute to the retention time, which hinders the uncertainty analysis. The traceability of DLS is also compromised by the lack of a suitable transfer standard for EVs, and the high number of parameters which contributes to the uncertainty of the measurement. In addition, DLS similarly to NTA, measures the hydrodynamic diameter of the particles.

### SAXS

The result presented in this manuscript is the first example for the use of SAXS in EV characterization. In general, SAXS characterization of nanoparticles can be made traceable using the oscillations that occur in the so-called Fourier regime (24, 44, 49) of the scattering curve. These oscillations are due to the fact that the wavelength of the used radiation is much smaller than the size of the observed particles, and distinguish SAXS from other ensemble techniques such as light scattering. However, for traceable size determination by SAXS, the sample needs to fulfil strict restrictions concerning monodispersity, shape and the complexity of the inner structure of the nanoparticle. EVs isolated using differential centrifugation cannot fit to these criteria due to their heterogeneity in size and the presence of non-vesicular contamination such as plasma proteins and lipoproteins. In case of a complex and heterogeneous sample, the interpretation of the scattering curve becomes an ill-posed problem, that is, different structural models can describe the same scattering curve. The erythrocyte EV sample studied in this manuscript belongs to the latter class, so the used model and the obtained parameters need to be justified by supplementary techniques. Using a model assumption, we have obtained the size distribution by fitting of a theoretical function to the measured scattering curve. A plausible size distribution was obtained, which resembles those obtained by the other methods. In addition, SAXS reveals the presence of plasma proteins in the sample that are not associated to the vesicles.

When comparing SAXS to the other techniques, first it should be emphasized that it needs a very specific infrastructure, which is available only at several synchrotron radiation laboratories worldwide. Examining EV samples on the other hand is on the border of the capabilities of this technique, as the EVs have heterogeneous size distribution by their nature. In addition, EVs belong to soft materials, hence their scattering power is low in comparison to solid nanoparticles. A concentration exceeding 10^11^ vesicles/mL is needed for this method, as the X-ray photons are scattered on electron density discontinuities, and the electron density contrast between the aqueous buffer and vesicles is relatively low. In summary, the 2 main challenges to reach the traceable characterization of EVs are the isolation of highly monodisperse and highly concentrated EV fractions, which are free of any non-vesicular contamination. Following the initial experiment presented in this study, a comprehensive and systematic study is being carried out as part of the METVES project (www.metves.eu). In METVES, SAXS will be used to characterize reference materials with similar properties to EVs and validate readily available laboratory scale instruments.

## Conclusion

In this study, the size distributions of an erythrocyte-derived EV sample obtained by several established (FF-TEM, NTA, RPS) and new techniques (SEC-DLS, SAXS) are presented. As demonstrated, all of the methods are capable of characterizing the mode diameter of the studied EV sample within small deviations, but traceability is not reached in either of the investigations. An appropriate uncertainty analysis is difficult in the case of the established methods in the field of EVs, such as NTA, RPS and DLS. An important practical aspect of NTA and RPS is that these methods are less laborious and need lower concentration of sample than SAXS and FF-TEM and also provide a fast characterization of the size of the vesicles. On the other hand, SAXS is capable of traceable characterization of solid nanoparticles in suspension. In order to also reach this goal in case of EVs, there is a need for highly monodisperse vesicle fractions with sufficient concentration for the SAXS analysis. As shown in this study, the combination of a separation step (SEC) directly linked to size detection technique (DLS) can help to overcome the limitations of the latter. Coupling of SAXS to SEC may represent a promising way towards traceable size determination of EVs, which, together with the development of reliable reference materials with similar properties to EVs, may facilitate standardization in the near future.
